# Identification of an IL-4-Inducible Gene Expressed in Differentiating Lymphocytes and Male Germ Cells

**DOI:** 10.1155/1990/94056

**Published:** 1990

**Authors:** Nasrin Nabavi, Michael J. Grusby, Patricia W. Finn, Debra J. Wolgemuth, Laurie H. Glimcher

**Affiliations:** 1 Department of Cancer Biology Harvard School of Public Health Boston Massachusetts 02115 USA; 2 Department of Genetics and Development and the Center for Reproductive Studies Columbia University College of Physicians and Surgeons 360 West 168th Street New York New York 10032 USA; 3 Department of Medicine Harvard Medical School Boston Massachusetts 02115 USA

## Abstract

Interleukin 4 (IL-4) is a cytokine that is involved in the differentiation of B and T lymphocytes. In this report, we describe the identification of a novel gene, N.52, which was cloned from the murine pre-B cell line R8205 grown in the presence of IL-4 for 48 hr. Although N.52 expression is detectable at low levels in unstimulated R8205 cells, the level of N.52 dramatically increases after only .4 hr exposure to IL-4 and remains at a high .level up to 48 hr. Although N.52 expression is low or absent in normal spleen B and T cells, its expression can be induced by the differentiation signals delivered by LPS in B cells and by Con A in T-cell hybrids. While N.52 mRNA is absent in all highly differentiated organs, it is detectable in stem cell harboring lymphoid tissues such as bone marrow, fetal liver, and thymus. Furthermore, N.52 mRNA is expressed at strikingly high levels in the testis, specifically in differentiating male germ cells. It is induced by differentiation signals triggered by the combination of cyclic AMP and retinoic acid in teratocarcinoma F9 cells. Taken together, these data suggest that N.52 is a developmentally regulated gene whose expression in cells of the immune and reproductive systems may be controlled by stimuli that induce differentiation.


*Developmental Immunology*, 1990, Vol. 1, pp. 19–30
Reprints available directly from the publisher
Photocopying permitted by license only

© 1990 Harwood Academic Publishers GmbH
Printed in the United Kingdom

### Identification of an IL-4-Inducible Gene Expressed in Differentiating Lymphocytes and Male Germ Cells

NASRIN NABAVI,[†] MICHAEL J. GRUSBY,[†] PATRICIA W. FINN,[†] DEBRA J. WOLGEMUTH[‡] and LAURIE H. GLIMCHER[*§]

[†]*Department of Cancer Biology, Harvard School of Public Health, Boston, Massachusetts 02115*
[‡]*Department of Genetics and Development and the Center for Reproductive Studies, Columbia University College of Physicians and Surgeons, 360 West 168th Street, New York, New York 10032*
[§]*Department of Medicine, Harvard Medical School, Boston, Massachusetts 02115*

Interleukin 4 (IL-4) is a cytokine that is involved in the differentiation of B and T lymphocytes. In this report, we describe the identification of a novel gene, N.52, which was cloned from the murine pre-B cell line R8205 grown in the presence of IL-4 for 48 hr. Although N.52 expression is detectable at low levels in unstimulated R8205 cells, the level of N.52 dramatically increases after only 4 hr exposure to IL-4 and remains at a high level up to 48 hr. Although N.52 expression is low or absent in normal spleen B and T cells, its expression can be induced by the differentiation signals delivered by LPS in B cells and by Con A in T-cell hybrids. While N.52 mRNA is absent in all highly differentiated organs, it is detectable in stem cell harboring lymphoid tissues such as bone marrow, fetal liver, and thymus. Furthermore, N.52 mRNA is expressed at strikingly high levels in the testis, specifically in differentiating male germ cells. It is induced by differentiation signals triggered by the combination of cyclic AMP and retinoic acid in teratocarcinoma F9 cells. Taken together, these data suggest that N.52 is a developmentally regulated gene whose expression in cells of the immune and reproductive systems may be controlled by stimuli that induce differentiation.

KEYWORDS: Interleukin-4 development, differentation, lymphocytes, male germ cells

### INTRODUCTION

Cellular differentiation involves a complex pattern of gene expression in response to extracellular and intracellular signals. External signals are delivered via binding of chemical mediators to specific receptors on the cell surface, and this binding results in the activation of gene programs in a selective, tissue-specific manner (Kelly et al., 1983; Krönke et al., 1985; Lau and Nathans, 1987; Ryder et al., 1988). Cells of the immune system are known to respond to a myriad of chemical mediators called cytokines or interleukins. One such cytokine, interleukin 4 (IL-4), is a T-cell and mast-cell derived peptide that regulates a broad spectrum of biological activities in several cell types (reviewed by Paul and Ohara, 1987). Receptors for IL-4 are expressed on most cells of hematopoietic lineage (Ohara and Paul, 1987; Park et al., 1987) as well as some nonhematopoietic cells (Lowenthal et al., 1988). In particular, IL-4 induces growth and differentiation of pre-B cells (Hofman et al., 1988), the hyperexpression of class II major histocompatibility complex (MHC) molecules (Noelle et al., 1984; Roehm et al., 1984; Polla et al., 1986) and Fc receptors for IgE on resting B lymphocytes (Conrad et al., 1987; Defrance et al., 1987), and, together with bacterial lipopolysaccharide (LPS), induces the differentiation of mature B cells into immunoglobulin-secreting plasma cells (Lutzker et al., 1988; Rothman et al., 1988). It is now clear that IL-4 also affects the development of T lymphocytes. IL-4 causes proliferation of antigen-stimulated

*Corresponding author.

T helper lymphocytes (Fernandez-Botran et al., 1986) and, together with phorbol esters (PMA), induces the differentiation of cytotoxic T lympocytes from intrathymic precursors (Palacios et al., 1987). IL-4 also synergyizes with another cytokine, interleukin 3, to generate mast cells from precursors in the bone marrow (Mosmann et al., 1986).

Similar to differentiation of the hematopoetic cells, development of the germ-cell lineage also involves progression through a highly specific series of differentiation events, in response to a poorly understood network of signals. The involvement of specific hormones and growth factors in these processes has been implicated, but the molecular mechanisms involved are unclear. Similarly, the molecular mechanisms by which IL-4 mediates the proliferative and differentiative programs of cells of the immune system are not well understood. In this report, we describe the isolation of a gene that is induced by IL-4 in a murine pre-B cell line. This gene, designated N.52, is also inducible in both B and T lymphocytes by differentiative and activating signals delivered by polyclonal activators such as LPS and Con A. The preferential expression of N.52 in bone marrow and thymus, lymphoid organs containing rapidly proliferating and differentiating cells, as well as its strikingly high level of expression in differentiating germ cells of the testis, suggest that N.52 is a developmentally regulated gene whose expression contributes to cellular growth and differentiation, particularly within the hematopoietic and germ-cell lineages.

### RESULTS

### Isolation of the N.52 cDNA

Earlier data from our laboratory demonstrated that IL-4 induces transcription of class II MHC genes in an Abelson virus transformed, IL-4 receptor-bearing pre-B cell line, R8205 (Polla et al., 1986). We sought to identify other genes induced by IL-4 using this IL-4-responsive cell line as a model system. A cDNA library was constructed from R8205 cells grown in the presence of IL-4 for 48 hr and approximately 60,000 independent recombinant phage plaques were screened in duplicate with a subtracted probe. The probe was obtained by synthesis of [$^{32}$P]-labeled cDNA from R8205 cells grown in the presence of IL-4, and subtraction of the cDNA with RNA derived from unstimulated R8205 cells. Positive clones (approximately 0.8%) identified by the first screening were rescreened by differential hybridization using cDNA probes prepared from R8205 cells grown in the presence or absence of IL-4. Close to 64% of the clones from this screening showed equivalent hybridization to both probes, 1% showed no hybridization to either probe, and 35% hybridized preferentially to the IL-4 stimulated probe. This last group contains genes that are specifically induced by IL-4. One clone from this group, N.52, hybridized selectively and strongly only to the induced probe. Northern blot analysis confirmed that 0.95-kb transcripts for N.52 are expressed at significantly higher levels in R8205 cells grown in IL-4 for 48 hr than in uninduced cells where only a variable low to faint signal was detected (Fig. 1A). The kinetics of induction of N.52 in R8205 cells by IL-4 is shown in Fig. 1B. N.52 levels increase within 4 hr of exposure to IL-4, continue to rise up to 24 hr and remain at a high level after 48 and 72 hr (not shown) growth in culture medium containing IL-4. Two additional higher molecular-weight transcripts are occasionally detected (see Fig. 5) and may represent partially spliced forms of N.52 nuclear RNA.

### Sequence Analysis of N.52 cDNA

The initially isolated partial cDNA clone contained an insert of 0.45 kb. Several additional clones obtained from screening four different cDNA libraries with the original insert or with a 5′ probe were sequenced and all found to contain at most 0.6 kb of 3′ sequence. Since it was possible that the secondary structure of the N.52 RNA was impeding the reverse transcriptase synthesis of cDNA, we next attempted to obtain more 5′ sequence by performing reverse transcription at 50°C to unfold secondary structure, followed by anchored polymerase chain reaction (APCR) using heat-stable Taq polymerase (Loh et al., 1989). Although this approach allowed us to obtain an additional 120 nucleotides that were enriched in G+C residues, we did not obtain a full-length cDNA clone. The nucleotide sequence of this partial cDNA contains a single open-reading frame, followed by a stop codon at nucleotide 361, and appears to extend to the 3′ end of mRNA since it contains a classic AAUAAA polyadenylation signal (Birnstiel et al., 1985) followed by a poly A tail (Fig. 2). This cDNA encodes a polypeptide of 120 amino acids (Fig. 2). A comparison of the DNA and amino acid sequence of clone N.52 with sequences in the GenBank (version 60, July 21, 1989) and Swiss

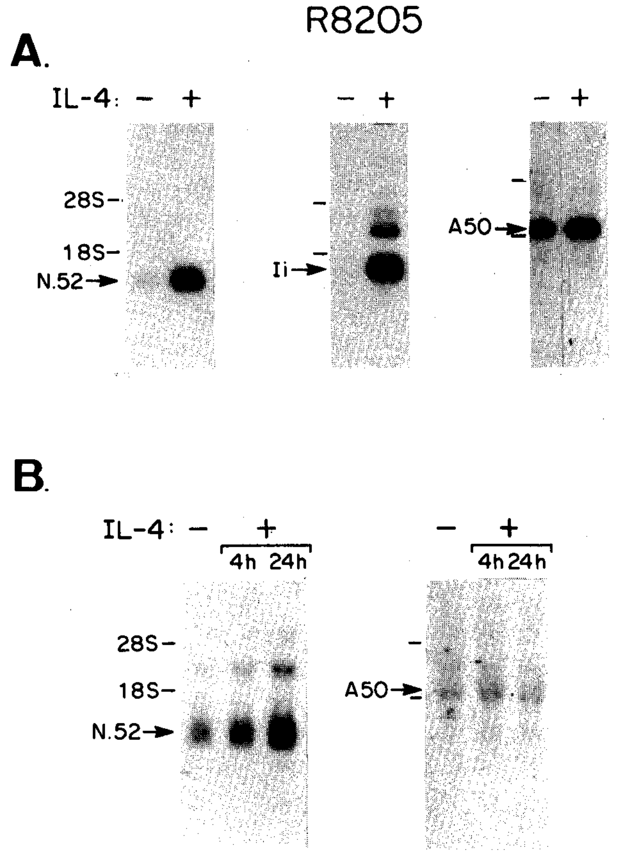

FIGURE 1. Induction of N.52 mRNA by IL-4 in R8205 cells. (A) Northern blot analysis of poly $A^+$ RNA prepared from R8205 cells; unstimulated (−) or cultured for 48 hr with IL-4 (+). Two $\mu$g of poly $A^+$ RNA were loaded in each lane and Nytran filters were hybridized with the N.52 cDNA probe (left panel; positions of 18 and 28S rRNA markers are indicated on the left). Subsequently, the filters were hybridized with Ii cDNA probe (Polla et al., 1986) to confirm the stimulatory effects of IL-4 (middle panel), and A50 cDNA (Nguyen et al., 1983) as a control for the amount of RNA loaded in each lane (right panel). (B) Kinetics of induction of N.52 mRNA by IL-4 in R8205 cells. Total cellular RNA was obtained from R8205 cells cultured in the absence or presence of IL-4 for varying time periods. Ten $\mu$g of total cellular RNA were loaded in each lane, and the blot was hybridized with N.52 cDNA probe. The probe was then stripped from the filter and hybridized to the A50 control cDNA probe to determine the level of RNA in each lane. In addition to the 0.95-kb N.52 message, higher molecular-weight transcripts are occasionally seen, and may represent nuclear RNA or partially spliced forms of N.52 transcript.

protein (version 10, July 21, 1989) databases, using computer programs, revealed no significant similarities. Further attempts to obtain additional 5′ sequence will focus on the analysis of a recently obtained genomic clone (Nabari, unpublished data).

### N.52 Expression Is Induced by Activating and Differentiating Stimuli

We have shown that IL-4 induces the expression of N.52 mRNA in a pre-B cell line known to be sensitive to IL-4-mediated signals. While IL-4 is known to provide a differentiation signal to B cells, it alone is a relatively weak activator of normal splenic B cells and requires costimulatory factors (Rabin et al., 1985; O'Garra et al., 1986; Snapper and Paul, 1987). It was therefore not surprising that IL-4 failed to increase N.52 transcripts in normal splenic B cells (not shown). In contrast, treatment of splenic B cells with bacterial lipopolysaccharide (LPS), a potent polyclonal B-cell activator, induced N.52 transcripts in athymic spleen cells, which consist predominantly of B cells (Fig. 3A). N.52 transcripts were induced after 4 hr of exposure to LPS and remained high after 18 hr, the latest time point tested. Because of the effect of LPS stimulation of B cells on levels of N.52 transcripts, it was of interest to determine whether stimulation of another lymphoid population, T cells, also affected N.52 expression. Northern analysis demonstrated that two antigen-specific T-cell hybrids (Glimcher and Shevach, 1982), created by fusing antigen-specific normal T cells with the BW5147 thymoma, lacked N.52 transcripts at baseline (Fig. 3B). The two T-cell hybrids were therefore treated with concanavalin A (Con A), which has been shown to induce the differentiation of splenic T cells into lymphokine-secreting cells (Farrar et al., 1980; Granelli-Piperno et al., 1984). Con A treatment of the two T hybrids induces high levels of N.52 mRNA (Fig. 3B), whereas similar treatment of splenic T cells failed to induce N.52 (not shown). These results suggest that splenic T cells may have passed the responsive stage for N.52 induction.

Since our data suggested that N.52 expression in lymphoid cells was inducible by differentiation stimuli, we were interested to see whether or not differentiation signals in nonlymphoid cells also induce N.52 expression. The F9 teratocarcinoma cell line is one such system in which differentiation can be triggered in vitro by a combination of cAMP and retinoic acid treatment (Strickland et al., 1980). Figure 3C demonstrates that these differentiation events coincide with the appearance of N.52 transcripts. Therefore, the induction of N.52 mRNA in response to differentiation signals is not limited to cells of the lymphoid lineage. Furthermore, these results suggest that N.52 expression accompanies active differentiation in different cell types.

```
  1 GAC GCG CGG CCC TCC CCG AGC GGT TGG CTG CAT ATA AGG CCC
    Asp Ala Arg Pro Ser Pro Ser Gly Trp Leu His Ile Arg Pro  14

 43 GCT CGC TTC TGG GCG TTA ACA TCT CCT GCC GCA GCC GCC CTA
    Ala Arg Phe Trp Ala Leu Thr Ser Pro Ala Ala Ala Ala Leu  28

 85 GAT TTG GAA TTC TAC ACT AAA GTC ATC ATG GGC GTT TTC CAG
    Asp Leu Glu Phe Tyr Thr Lys Val Ile Met Gly Val Phe Gln  42

127 ATA TTG ATG AAG AAT AAG GAA CTC ATT CCT TTG GCG TTT TTT
    Ile Leu Met Lys Asn Lys Glu Leu Ile Pro Leu Ala Phe Phe  56

169 ATA AGC GTG GCC GCC ACC GGA GCC ACA TCT TTC GCT TTG TAT
    Ile Ser Val Ala Ala Thr Gly Ala Thr Ser Phe Ala Leu Tyr  70

211 GCG TTG AAA AAA ACC GAT GTG GTT ATT GAT CGG AAA AGA AAC
    Ala Leu Lys Lys Thr Asp Val Val Ile Asp Arg Lys Arg Asn  84

253 CCA GAG CCT TGG GAA ATG GTG GAT CCT ACT CAA CCC CAA AAG
    Pro Glu Pro Trp Glu Met Val Asp Pro Thr Gln Pro Gln Lys  98

295 CTT ATA ACC ATC AAC CAG CAA TGG AAG CCC GTT GAG GAG CTG
    Leu Ile Thr Ile Asn Gln Gln Trp Lys Pro Val Glu Glu Leu  112

337 CAA AAA GTC CGG AGG GCA ACC AGA TGA TTG CTC ACC ACT CCT
    Gln Lys Val Arg Arg Ala Thr Arg •••                      120

379 CTC TTC CAA AGA ACA CTC TAT GAA TCT AGT GGA GAC ATT TCT
421 GCA CAA ACT AGA TGT TGA TGC CAG TGT GCG GAA ATG CTT CTG
463 CTA CAT TTG TAG GGT TTG CCT GCA TTC TTT GGA TCC TGC ATT
505 AGC AAG TGA AGG TAG CAC ATA GTC TAA AAT AGT TTT CTG TGT
547 TTA TTG GTG TAA ATT TCA ATT TTA CAG TTG AAA TTT TAT GTT
589 TGT GAT GCT TGG ATA TTT TCC TTG AAA TGT ATA AAC ATG TAA
631 AAA TTA GAT TAC TGC CTG TAA TAA AAT AAT TCG ATG ACT ATC
673 TGT AAA ACA TGA AAA AAA AAA AAA A
```

FIGURE 2. Nucleotide and deduced amino acid sequences of N.52 partial cDNA. The coding strand contains an open reading frame of 120 amino acids terminating by a stop codon at position 361. The cDNA contains a consensus polyadenylation signal at position 650 (boxed) followed by a poly A tail.

### N.52 Expression in Normal Mouse Tissue

RNA extracted from freshly isolated mouse tissue was examined by Northern blot analysis (Fig. 4). N.52 expression was undetectable in highly differentiated organs such as brain, heart, lungs, liver, and kidney. In contrast, N.52 mRNA was present in thymus, bone marrow, and day-17 fetal liver, organs harboring actively developing and differentiating cells.

Most striking, however, was the extremely high level of expression of N.52 in testis, an organ composed both of differentiating germ cells and somatic cells. N.52 mRNA was not detectable in ovary (not shown). To investigate the relative level of expression of N.52, testis RNA was hybridized with the Hox.-1.4 probe, a homeobox-containing gene expressed abundantly in the adult mouse testis (Wolgemuth et al., 1986, 1987) (Fig. 4, right panel). The results shown in Fig. 4 demonstrate that N.52 transcripts are significantly more abundant in testis than Hox-1.4 transcripts. Subsequent hybridization of these blots to control probe, A50, indicated that comparable levels of RNA were present in each lane. The expression of N.52 in testis is parallel to the expression of the homeobox gene Hox-1.4 (Wolgemuth et al., 1986), except that the level of expression of N.52 is much higher than Hox-1.4. This may be due to the greater potency of the N.52 promoter/enhancer sequences in germ cells, although other possibilities such as RNA stability effects cannot be ruled out.

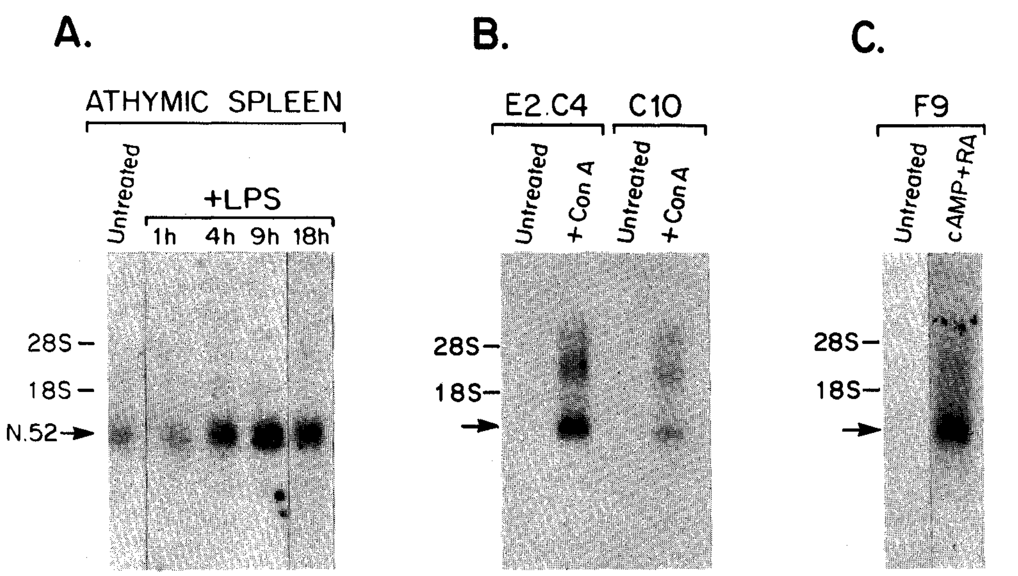

FIGURE 3. Induction of N.52-specific mRNA by differentiation stimuli. (A) Northern blot analysis of 15 μg total cellular RNA prepared from athymic mouse spleen cells cultured in the absence or presence of 50 μg/ml LPS for different time periods. (B) Northern blot analysis of 15 μg RNA prepared from T-cell hybrids E2C4 and C10 cultured in medium alone or in the presence of 20 μg/ml Con A for 24 hr. (C) Northern blot analysis of F9 tratocarcinoma cell line grown undifferentiated or differentiated with cAMP and retinoic acid for 3 days. All blots (A, B, and C) were subsequently hybridized to A50 control probe.

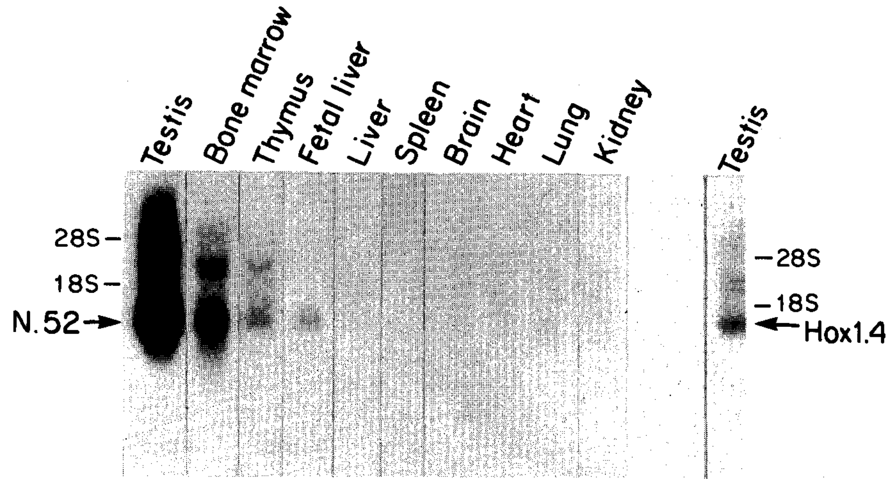

FIGURE 4. Tissue distribution of N.52 expression [$^{32}$P]-labeled N.52 probe was hybridized to 25 μg total RNA prepared from different tissues. Exposure time was 18 hr at −70°C. For comparison of the level of N.52 mRNA with Hox−1.4, a gene with known expression in testis, the lane containing testis RNA was subsequently hybridized with the Hox−1.4 (0.8 kb Pst/Hind III genomic fragment (Wolgemuth et al., 1987). Exposure was 3 days at −70°C. The autoradiographs are not lined up, as the Hox−1.4 mRNA is 1.3 to 1.4 kb in length. The blots were subsequently hybridized to A50 control probe.

### Expression of N.52 in Testis Is Limited to Germ Cells

The extremely high level of N.52 transcripts in testis prompted experiments to determine which testicular cell type(s) is involved in this expression. Two different and complementary approaches were used. Both neonatal and adult testes have a full complement of somatic cells, which include Leydig, Sertoli, peritubular, and macrophagelike cells. Germ-cell development in the testis is not complete, however, until approximately 4 weeks of age. We compared the level of N.52 transcripts in testes from athymic mice and from normal C3H mice, day 2, day 14, and adult (4 and 8 weeks) animals (Fig. 5A). In the athymic mice, N.52 transcripts were not detected in immature testes (day 2 postnatal), were present at very low levels in developing testes (day 14), and at very high levels in adult testes. In C3H mice, levels of N.52 transcript were already almost maximal by day 14. As discussed in what follows, athymic mice exhibit delayed development in comparison with

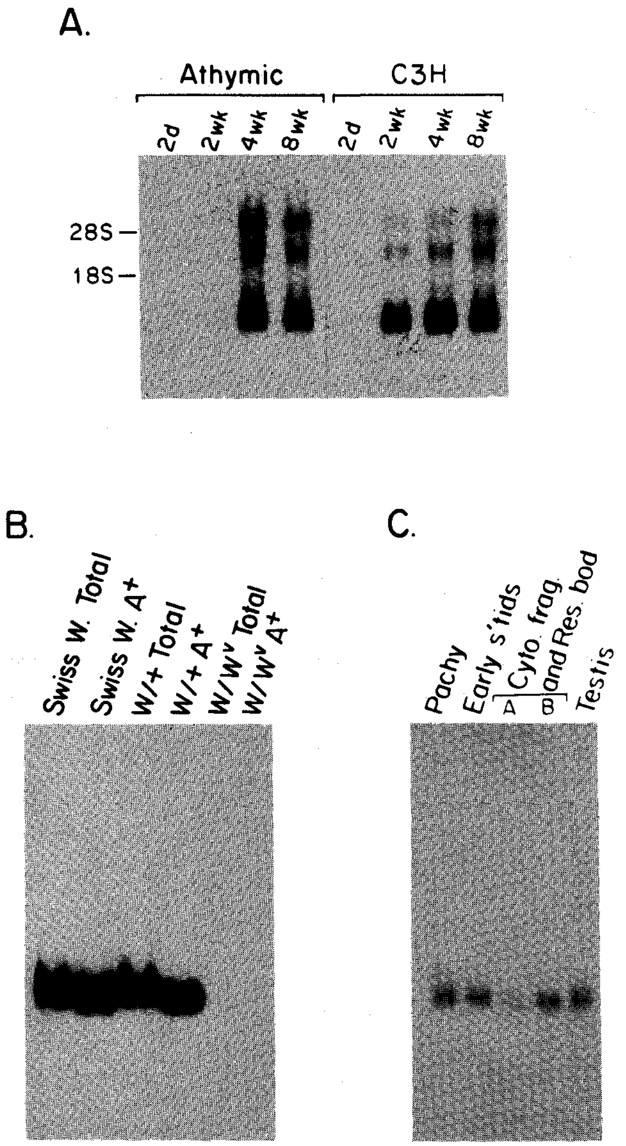

FIGURE 5. Expression of N.52 in mouse testis. (A) Northern blot analysis of total RNA from athymic or C3H mice testis from age of 2 days to 8 weeks probed with [$^{32}$P]-labeled N.52 cDNA probe. (B) Either total (20 $\mu$g) or poly A$^+$ (2.5 $\mu$g) RNA was Swiss Webster (Swiss W) wild type, heterozygous (w/$^+$) and homozygous (W/W$^v$) mutant germ-cell-deficient mice testes probed with [$^{32}$P]-labeled N.52 cDNA. Exposure 2 days. (C) Northern blot analysis of total RNA ( ~ 30 $\mu$g per lane except as noted) isolated from three different spermatogenic cell populations including pachytene spermatocytes, early spermatids, and the residual bodies and cytoplasmic fragments. Samples A and B refer to two different concentrations of RNA ( ~ 30 and 60 $\mu$g respectively). Subsequently, the blot was hybridized with an actin cDNA probe. Exposure 7 days. The relatively low level of signal is presumably due to the fact that this blot had been hybridized and stripped of probe several times.

normal animals, and this developmental retardation may extend to germ-cell maturation. This experiment suggested that the expression of N.52 was likely to be correlated with differentiating germ cells, and that the appearance of N.52 transcripts coincided with the beginning of meiosis.

To test this more rigorously, RNAs were prepared from testes of a mutant strain of mice, W/W$^v$, whose testes are germ-cell-deficient. Gonads from mice homozygous for the W alleles (W/W$^v$) are virtually devoid of germ cells, and histological examination of W/W$^v$ testes reveals normal somatic cells but few or no identifiable germ cells (Coulumbre and Russell, 1954). RNA was also isolated from testes of sexually mature heterozygous siblings W/$^+$, and from adult Swiss Webster mice. Total and poly A$^+$ RNA prepared from testicular tissue of the fertile animals contained N.52 transcripts (Fig. 5B, first four lanes). In contrast, no N.52 transcripts were detected in either total or poly A$^+$ RNA from the germ-cell-deficient W/W$^v$ testes (Fig. 5B, last two lanes). These results indicate that the expression of N.52 transcripts in testes is germ-cell-specific since all of the somatic cell types are present in the testes of homozygous W/W$^v$ animals.

The quantitative differences in expression of N.52 transcript between immature and mature testis (Fig. 5A, compare day 2 to 4 weeks) suggest that its appearance correlates with meiotic stages of spermatogenesis. To determine the developmental stage at which the N.52 transcript appears, cells from adult testis were purified by sedimentation at unit gravity (Wolgemuth et al., 1985). RNAs were isolated from three different cellular populations, including the meiotic prophase spermatocytes (predominantly in pachytene stage), the early postmeiotic spermatids, and a fraction that included residual bodies and cytoplasmic fragments of elongating spermatids, and were probed for expression of N.52 mRNA (Fig. 5C, first four lanes). N.52 was not expressed in testes that contain only premeiotic germ cells (see Fig. 4A, 2d postnatal). N.52 transcript was present in germ cells in meiotic prophase (pachytene) and in cells that were further advanced (spermatids) in the developmental pathway of spermatogenesis. No striking differences in levels of N.52 were seen in the different stages, including cytoplasmic fragments and residual bodies (Fig. 5C), when RNA loading in each lane was normalized. These data suggest that N.52 expression is likely to be induced by differentiation signals at the onset of meiosis and remains high during all the stages of germ-cell development.

### The N.52 Gene Is Highly Conserved

Genomic Southern blot analysis of mouse DNA digested with a panel of restriction endonucleases probed with a 0.6-kb cDNA insert (Fig. 6) revealed a fairly simple pattern consistent with the presence of a single copy gene. A cross-hybridizing species in human DNA remained easily detectable even under stringent hybridization and wash conditions (Fig. 6), suggesting some degree of evolutionary conservation of N.52 across species.

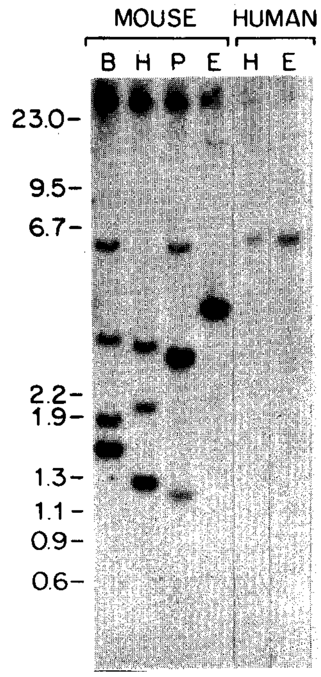

FIGURE 6. Genomic Southern blot analysis of N.52 in mouse and human DNA. DNAs were digested with (B) BamHI, (H) Hind H III, (P) PstI, or (E) EcoRI, hybridized, and washed under stringent conditions, as described in the experimental procedures. Phage λ Hind III digested DNA size markers are indicated.

### DISCUSSION

Despite the multiplicity of effects of IL-4 on various cell types, very little is known about the molecular mechanisms by which IL-4 exerts these effects. Those genes that are known to be induced by IL-4 include class II MHC (Polla et al., 1986), Fc epsilon receptor (Hudak et al., 1987), Thy-1 (Snapper et al., 1988), and mouse pancreatic lipase (Grusby, unpublished data). In this report, we describe the isolation of an IL-4 inducible gene, designated N.52, by differential and subtractive hybridization screening of a murine pre-B cell cDNA library.

N.52 is a novel gene that demonstrates several interesting features. First, it is inducible by IL-4, which is known to initiate a differentiation program in pre-B cells (Hofman et al., 1988) and by other polyclonal activators such as Con A and LPS that trigger differentiation in lymphoid cells (Granelli-Piperno et al., 1984; Rothman et al., 1988). Second, it is expressed in bone marrow, thymus, testis, and fetal liver, organs harboring rapidly growing and differentiating stem cells. Third, the very high level of N.52 expression in developing germ cells, and its inducibility in response to differentiation signals in F9 cells in lymphoid cells demonstrate that N.52 expression accompanies differentiation events in widely disparate cell types.

Mammalian cells respond to mitogenic and differentiating stimuli by sequential activation of selected genes. Immediate early response genes such as the *c-fos*, *c-jun*, and *myc* protooncogenes are transcriptionally activated within minutes after exposure to the relevant stimuli (Kelly et al., 1983; Greenberg and Ziff, 1984; Ryder et al., 1988). Activation of this set of genes is followed by activation of a second set (Kwon et al., 1987) (such as transferrin and interleukin 2 receptor genes) (Krönke et al., 1985) whose transcripts accumulate later and remain high for more extended periods. The kinetics of N.52 induction in cells of B lineage in response to external stimuli resemble the latter group. Although N.52 transcripts are detectable in unstimulated R8205 pre-B cells, exposure to IL-4 begins to increase steady-state mRNA levels by 4 hr. N.52 transcripts increase and are maintained at maximal level for up to 72 hr after IL-4 exposure. These kinetics are similar to the induction of the class II MHC genes by IL-4 in both the R8205 pre-B cell line and in normal splenic B cells (Noelle et al., 1986; Polla et al., 1986). Another similarity between N.52 and class II MHC expression in the B lineage is the absence of expression of both these gene products in myelomas, a terminally differentiated B cell state (Nabavi, unpublished data).

Activation and differentiation of lymphoid cells is modulated through complex interactions of numerous extracellular stimuli, which may synergize or interfere with IL-4 mediated signals, depending on cell type and developmental stage (Ohara and Paul, 1987; Paul and Ohara, 1987). These stimuli include LPS, which causes blast transformation of resting B cells and drives them to Ig production (Snapper and Paul, 1987; Lutzker et al., 1988; Rothman et al., 1988), and Con A, which activates expression of some genes, including lymphokines and lymphokine receptors in resting T cells (Farrar et al., 1980; Krönke et al., 1985; Burd et al., 1987; Kwon et al., 1987). Some other stimuli include PMA, αIgM, and lymphokines (Farrar et al., 1980; Granelli-Piperno et al., 1984; Krönke et al., 1985; Rabin et al., 1985). N.52 expression is induced by a subset of these activation and differentiation signals, including IL-4, LPS, and Con A, but not by PMA (not shown). The ability of a particular stimulus to induce N.52 expression varies with cell type and developmental stage, as LPS, but not IL-4, can induce N.52 in resting B cells. IL-4, however, can induce N.52 in R8205 cells, which are transformed cells in the pre-B cell stage of development. Similarly, Con A induced N.52 expression in T hybrids, but not in splenic T cells (not shown). It is possible that splenic T cells have already passed the responsive stage for N.52 induction. Alternatively, Con A may induce N.52 in the T hybrids possibly by acting upon the BW5147 thymoma fusion partner. It will be interesting to identify the critical stimuli necessary for N.52 induction at the various stages of B- and T-cell development.

The overlapping expression of N.52 within the lymphoid and reproductive compartments is not surprising since this has been shown for several gene products. Class II MHC antigens and CD4-like molecules have been reported on male germ cells (Ashida and Scofield, 1987; Bishara et al., 1987). In addition, an IL-1α-like soluble factor has been found in testicular interstitial fluid (Gustafsson et al., 1988). Other lines of evidence also link the immune and reproductive systems physiologically, via direct or indirect hormonal interactions. Differences in humoral and cell-mediated immune responses in male and female animals have been attributed to the effects of the sex hormones on the immune system (Paavonen et al., 1981). The effects of sex hormones in thymic development have been demonstrated; for example, castration of both male and female animals results in spleen and thymus hyperplasia and an increase in peripheral lymphocyte counts (Eidinger and Garrett, 1972; Allen et al., 1984). Reciprocal effects of the thymus on reproductive development have also been noted (Strich et al., 1985). Congenitally athymic mice show reproductive defects, and these defects can be corrected by injection of thymosin or by thymus grafts (Strich et al., 1985). In view of the thymus–reproductive system interaction, our observation of the lag time in the expression of N.52 in testes of 2-week-old athymic mice compared to the age-matched normal animals is interesting. However, the significance of this observation is not clear at present.

The process of spermatogenesis involves a series of cellular differentiation steps in which spermatogenic stem cells undergo functional and morphological specialization to form spermatozoa. Identification of stage-specific gene products is of particular interest in understanding this complex pathway of cellular differentiation. Some of these gene products are expressed at specific stages and thus presumably involved in basic cellular functions underlying morphological changes that occur during spermatogenesis, whereas others, such as the protooncogenes c-*abl* (Ponzetto and Wolgemuth, 1985), c-*mos* (Goldman et al., 1987), *pim*-1 (Sorrentino et al., 1988), and *int*-1 (Schackleford and Varmus, 1987), have been implicated in postmeiotic stages of spermatogenic differentiation. Several nuclear protooncogenes such as c-*myc* and c-*fos*, which are activated during germ-cell differentiation, are also induced by different stimuli during lymphocyte differentiation (Klemsz et al., 1989; Wolfes et al., 1989). Protooncogenes c-*mos* and c-*raf* are members of the serine and threonine kinase families, respectively, with overlapping expression in somatic and germ cells (Wolfes et al., 1989). Although the function of the N.52 gene product is not yet known, its induction by different stimuli (sex hormones, lymphokines, polyclonal activators) that act through different signal transduction pathways suggests that it may function in a common final pathway of cellular growth and differentiation. Further, its presence in such different cell types suggests that phenotypically and ontologically distinct cells may utilize overlapping molecular pathways for differentiation.

### MATERIALS AND METHODS

### cDNA Synthesis and Cloning

R8205 cells were grown in the presence of 800 U of rIL-4/ml (Immunex Corp., Seattle, Washington) for 48 hr. R8205 cells stimulation by IL-4 was confirmed by surface class II MHC expression, as determined by flow cytometry analysis (Polla et al., 1986). Poly $A^+$ RNA was prepared by oligo dT cellulose chromatography of total RNA, isolated by the guanidine isothiocyanate method (Chirgwin et al., 1979). First-strand cDNA was synthesized from 4 $\mu$g of poly $A^+$

RNA with a slight modification of the method described by Huynh et al. (1985) using oligo (dT) primer (Promega) and Avian myeloblastosis virus reverse transcriptase (Life Sciences, Saint Petersburg, Florida). Second-strand cDNA was synthesized using DNA polymerase I (New England Biolabs) and RNase H (Promega Biotec, Madison, Wisconsin). After methylation at EcoRI sites, cDNA was blunted, and ligated to EcoRI linkers. Free linkers were removed, and double-stranded DNA was size fractionated by chromatography on Sepharose CL-4B (Pharmacia), as described by Klickstein and Neve (1987). cDNA fragments greater than 500 bp were ligated into the EcoRI site of λgt10 (Stratagene, La Jolla, California). The recombinant clones were packaged in vitro using Gigapack (Stratagene) according to the manufacturer's protocol. Approximately $1.5 \times 10^6$ recombinant clones were obtained. Subcloning of cDNA inserts into pBluescript (Stratagene) was performed according to standard methods (Maniatis et al., 1982).

### Screening of cDNA Library

Approximately 60,000 plaque-forming units (*pfu*) from the λgt10 cDNA library were screened. About 2,000 *pfu* were inoculated per 150-mm diameter petri dish. After overnight growth, duplicate lifts were made from each plate onto nitrocellulose. The filters were fixed according to standard protocol (Maniatis et al., 1982) and prehybridized at 65°C in 4×SSC, 33% Dextran Sulfate, 2×Denhardt's and 100 μg denatured salmon sperm DNA for 4 hr. Hybridization was performed in a fresh buffer that contained $0.5 \times 10^6$ cpm/ml subtracted cDNA probe. Subtracted cDNA probes were synthesized as described (Davis, 1986). The single-stranded cDNA from IL-4 stimulated R8205 cells was radiolabeled to approximately $2.4 \times 10^8$ cpm per μg with [$\alpha^{32}$P]dCTP, and subtracted with a ten-fold excess of poly $A^+$ mRNA from unstimulated R8205 cells. The cDNAs that remained single-stranded after hybridization to $R_0{}^t$ of 1200 (nucleotide per liter) ×sec were isolated by hydroxylapatite chromatography. The subtracted probe constituted 5–10% of the total column input (~$1.8 \times 10^7$ cpm). After hybridization at 65°C for 72 hr, the filters were washed once in 2×SSC+0.5% SDS at room temperature for 20 min, followed by two washes in the same solution at 55°C for a total of 40 min. The final wash was done in 0.2×SSC+0.1% SDS at 55°C for 10 min. The filters were exposed to Kodak X-AR film at −70°C for 48 to 72 hr. The primary positive plaques (~500 *pfu*) were screened differentially by means of hybridization of each set of duplicate replica filters to cDNA probes made from either IL-4-stimulated or -unstimulated R8205 cells. The differential hybridization was performed in 48% formamide, 5×SSC, 10 mM Tris-HCl (pH 7.6), 1×Denhardt's, 1% SDS, 10% Dextran sulfate, and 100 μg/ml denatured salmon sperm DNA at 42°C containing $1 \times 10^6$ cpm/ml of each relevant cDNA probe. Equal numbers of cpm from each probe were hybridized to each set of duplicate filters. The filters were washed twice with 2×SSC+0.1% SDS at room temperature and twice with 0.2×SSC+0.1% SDS at 65°C. Exposure of the filters to Kodak X-AR film was done at room temperature for 24 to 48 hr.

### DNA Sequence Analysis

The N.52 cDNA fragments were subcloned into the phagemid pBluescript (Stratagene) in both orientations. The nucleic acid sequence of both strands was determined by the dideoxynucleotide-chain-termination method (Sanger, 1977), using a combination of synthetic oligonucleotide primers. The sequence of the GC-rich regions was determined by chemical sequencing (Maxam and Gilbert, 1980). The nucleotide sequence, as well as the predicted protein sequence, was compared with sequences in the GenBank and Swiss protein databases using the Bionet System.

### RNA Preparation and Northern Blot Analysis

Total cellular RNA from different cell lines was extracted by lysing the cells in 4 M guanidinium isothiocyanate. RNA from mouse tissue was prepared by homogenizing freshly isolated tissue in 4 M guanidinium isothiocyanate using a polytron homogenizer (Brinkman, Westbury, New York) followed by $CsCl_2$ gradient centrifugation (Chirgwin et al., 1979). RNA from $W^v$/W germ-cell-deficient mice and normal littermates and RNA from enriched populations of germ cells in various stages of differentiation were prepared essentially as described previously (Wolgemuth et al., 1985). Lipopolysaccharide stimulation of athymic mice spleen cells was done by culturing the cells in medium containing 50 μg/ml LPS for different time periods. T-cell hybrids were cultured unstimulated or in the presence of 20 μg/ml Con A for 24 hr before RNA was extracted from the cells. Total RNA from F9 cells grown unstimulated or treated with $5.0 \times 10^{-7}$ M retinoic acid (RA) and 0.5 mM dibutyryl cyclic AMP (cAMP) was the gift of Dr. Richard Niles (Boston University School of Medicine). Poly $A^+$ RNAs were selected by 1×passage through oligo [dT] cellulose (New England Biolabs) columns, by standard methods (Maniatis et al., 1982). Total RNA (10-20 $\mu$g) or poly $A^+$ RNA (1-2.5 $\mu$g) was denatured in 17% formaldehyde, 33% formamide, and size fractionated on a 1.3% agarose gel containing 1.6% formaldehyde. RNA was transferred to Nylon filters, Nytron (Schleicher & Schuell) in 20×SSC overnight. RNA blots of mutant or fractionated cell samples were separated on 0.8% gels and transferred to GeneScreen Plus. Filters were baked at 80°C under vacuum for 2 hr, then hybridized to a nicktranslated (Rigby et al., 1977) N.52 cDNA probe or to control probe A50. The hybridization buffer, described in a secondary screening of the cDNA library, was used, except that 1 to $3 \times 10^6$ cpm/ml of [$^{32}$P]-labeled probe was included. Control probe A50, a rat gene containing sequences for a constitutively expressed "housekeeping" mRNA of unknown identity (Nguyen et al., 1983), was used to determine the amount and integrity of RNA loaded in each lane. Invariant chain (Ii), a class II MHC associated cDNA probe, was used as a positive control to assess the degree of stimulation by IL-4 in R8205 cells (Polla et al., 1986). Blots were hybridized for 18-24 hr at 42°C and washed as essentially described in a secondary screening of the cDNA library except that filters hybridized to A50 were washed in 2×SSC, 0.1% SDS at 65°C.

### Genomic Southern Blot Analysis

Southern analysis was performed as described (Maniatis et al., 1982). Ten $\mu$g of genomic DNA was digested with EcoR1, BamHI, Hind III, or PstI at 37°C for 4 hr. Digested DNA was electrophoresed onto a 0.9% agarose gel overnight and blotted onto nitrocellulose filters. Filters were baked at 80°C under vacuum, and hybridized to the nick-translated probe as described in Northern analysis.

### ACKNOWLEDGMENTS

We are grateful to Dr. Richard Nile for providing us with F9 teratocarcinoma cells and RNA. We also thank Dr. Lionel Ivashkiv for helpful discussions and critical reading of the manuscript, Dr. Margaret Baer for help with this work, and Ms. Linda Blood for secretarial assistance.

NN is supported by a postdoctoral fellowship from the Cancer·Research Institute, MJG by an Arthritis Foundation Fellowship, and PWF by a Pfizer Postdoctoral Fellowship. LHG is a scholar of the Leukemia Society of America. This work was supported by NIH grants GM36864 (LHG), HL02054-3 (PWF), and HDO 5077 (DJW)

*(Received November 18, 1989)*

*(Accepted December 6, 1989)*

### REFERENCES

Allen L.S., McClure J.E., Goldstein A.L., Barkley M.S., and Michael S.D. (1984). Estrogen and thymic hormone interactions in the female mouse. J. Reproduc. Immunol. **6:** 25–37.

Ashida E.R., and Scofield V.L. (1987). Lymphocyte major histocompatibility complex-encoded class II structures may act as sperm receptors. Proc. Natl. Acad. Sci. USA **84:** 3395–3399.

Birnstiel M.L., Busslinger M., and Strub K. (1985). Transcription termination and 3' processing: the end is in site! Cell **41:** 349–359.

Bishara A., Oksenberg J.R., Frankel G., Margalioth E.I.J., Persitz E., Nelken D., Friedmann A., and Brautbar C. (1987). Human leukocyte antigens (HLA) class I and class II on sperm cells studied at the serological, cellular, and genomic levels. Am. J. Reproduc. Immunol. Microbiol. **13:** 97–103.

Burd P.R., Freeman G.J., Wilson S.D., Berman M., DeKruyff R., Billings, P.R., and Dorf M.E. (1987). Cloning and characterization of a novel T cell activation gene. J. Immunol. **139:** 3126–3131.

Chirgwin J.M., Przybyla A.E., MacDonald R.J., and Rutter W. J. (1979). Isolation of biologically active ribonucleic acid from sources enriched in ribonuclease. Biochemistry **18:** 5294–5299.

Conrad D.H., Waldschmidt T.J., Lee W.T., Rao M., Keegan A.D., Noelle R.J., Lynch R.G., and Kehry M.R. (1987). Effect of B cell stimulatory factor-1 (interleukin 4) on $Fc_\varepsilon$ and $Fc_\gamma$) receptor expression murine B lymphocytes and B cell lines. J. Immunol. **139:** 2290–2296.

Coulumbre J.L., and Russell E.S. (1954). Analysis of the pleiotropism at the w-Locus in the mouse. J. Exp. Zool. **126:** 277–291.

Davis M.M. (1986). Subtractive cDNA hybridization and the T-cell receptor genes. *In:* Handbook of experimental immunology. (Oxford: Blackwell Scientific Publications), pp. 76.1–76.13.

Defrance T., Aubry J.P., Rousset F., Vanbervliet B., Bonnefoy J.Y., Arai N., Takebe Y., Yokota T., Lee F., Arai K., de Vries, J., and Banchereau J. (1987)). Human recombinant interleukin 4 induces $Fc_\varepsilon$ receptors (CD23) on normal humn B lymphocytes. J. Exp. Med. **165:** 1459–1467

Eidinger D., and Garrett T.J. (1972). Studies of the regulatory effects of the sex hormones on antibody formation and stem cell differentiation. J. Exp. Med. **136:** 1098–1116.

Farrar J.J., Fuller-Farrar J., Simon P.L., Hilfiker M.L., Stadler B.M., and Farrar W.L. (1980). Thymoma production of T cell growth factor (interleukin 2). J. Immunol. **125:** 2555–2558.

Fernandez-Botran R., Sanders V.M., Oliver K.G., Chen Y.-W., Krammer P.H., Uhr J.W., and Vitetta E.S. (1986). Interleukin 4 mediates autocrine growth of helper T cells after antigenic stimulation. Proc. Natl. Acad. Sci. USA **83:** 9689–9693.

Glimcher L.H., and Shevach E.M. (1982). Production of autoreactive I region-restricted T cell hybridomas. J. Exp. Med. **156:** 640–645.

Goldman D.S., Kiessling A.A., Millette C.F., and Cooper G.M. (1987). Expression of c-*mos* RNA in germ cells of male and female mice. Proc. Natl. Acad. Sci. USA **84:** 4509–4513.

Granelli-Piperno A., Inaba K., and Steinman R.M. (1984). Stimulation of lymphokine release from T lymphoblasts. J. Exp. Med. **160:** 1792–1802.

Greenberg M.E., and Ziff E.B. (1984). Stimulation of 3T3 cells induces transcription of the c-*fos* proto-oncogene. Nature **311:** 433–438.

Gustafsson K., Söder O., Pöllänen P., and Ritzèn E.M. (1988). Isolation and partial characterization of an interleukin-1-like factor from rat testis interstitial fluid. J. Reproduc. Immunol. **14:** 139–150.

Hofman F.M., Brock M., Taylor C.R., and Lyons B. (1988). IL-4 regulates differentiation and proliferation of human precursor B cells J. Immunol. **141:** 1185–1190.

Hudak S.A., Gollnick S.O., Conrad D.H., and Kehry M.R. (1987). Murine B-cell stimulatory factor 1 (interleukin 4) increases expression of the Fc receptor for IgE on mouse B cells. Proc. Natl. Acad. Sci. USA **84:** 4604–4610.

Huynh T.V., Young R.A., and Davis R.W. (1985). Construction and screening cDNA libraries in $\lambda$gt10 and $\lambda$gt11. *In:* DNA cloning: a practical approach, vol. 1, Glover D., Ed. (Arlington, VA: IRL), pp. 49–78.

Kelly K., Cochran B.H., Stiles C.D., and Leder P. (1983). Cell-specific regulation of the c-*myc* gene by lymphocyte mitogens and platelet-derived growth factor. Cell **35:** 603–610.

Klemsz M.J., Justement L.B., Palmer E., and Cambier J.C. (1989). Induction of c-*fos* and c-*myc* expression during B cell activation by IL-4 and immunoglobulin binding ligands. J. Immunol. **143:** 1032–1039.

Klickstein L.B., and Neve R.L. (1987). Methylation and addition of linkers to double-stranded cDNA. *In:* Current protocols in molecular biology, Ausubel M., Brent R., Kingston R.E., Moore D.D., and Smith A.J. Eds. (New York: John Wiley and Sons), pp. 5.6.1–5.6.8.

Krönke M., Leonard W.J., Depper J.M., Arya S.K., Wong-Staal F., Gallo R.C., Waldmann T.A., and Greene W.C. (1984). Cyclosporin A inhibits T cell growth factor gene expression at the level of mRNA transcription. Proc. Natl. Acad. Sci. USA **81:** 5214–5218.

Krönke M., Leonard W.J., Depper J.M., and Greene W.C. (1985). Sequential expression of genes involved in human T lymphocyte growth and differentiation. J. Exp. Med. **161:** 1593–1598.

Kwon B.S., Kim G.S., Prystowsky M.B., Lancki D.W., Sabath D.E., Pan J., and Weissman S.M. (1987). Isolation and initial characterization of multiple species of T-lymphocyte subset cDNA clones. Proc. Natl. Acad. Sci. USA **84:** 2896–2900.

Lau L.F., and Nathans D. (1987). Expression of a set of growth-related immediate early genes in BALB/c 3T3 cells: coordinate regulation with c-*fos* or c-*myc*. Proc. Natl. Acad. Sci. USA **84:** 1182–1186.

Loh E.Y., Elliott J.F., Cwirla S., Lanier L.L., and Davis M.M. (1989). Polymerase chain reaction with single-sided specificity: analysis of T cell receptor $\delta$ chain. Science **243:** 217–220.

Lowenthal J.W., Castle B.E., Christiansen J., Schreurs J., Rennick D., Arai N., Hoy P., Takebe Y., and Howard M. (1988). Expression of high affinity receptors for murine interleukin 4 (BSF-1) on hemopoietic and nonhemopoietic cells. J. Immunol. **140:** 456–464.

Lutzker S., Rothman P., Pollock R., Coffman R., and Alt F.W. (1988). Mitogen- and IL-4-regulated expression of germ-line Ig $\gamma$2b transcripts: evidence for directed heavy chain class switching. Cell **53:** 177–184.

Maniatis T., Fritsch E.F., and Sambroock J. (1982). Molecular cloning: a laboratory manual. (Cold Spring Harbor, NY: Cold Spring Harbor Laboratory).

Maxam A.L., and Gilbert W. (1980). Sequencing end-label DNA with base specific chemical cleavages. Methods Enzymol. **65:** 499–559.

Mosmann T.R., Bond M.W., Coffman R.L., Ohara J., and Paul W.E. (1986). T-cell and mast cell lines respond to B-cell stimulatory factor 1. Proc. Natl. Acad. Sci. USA **83:** 5654–5658.

Nguyen H.T., Medford R.M., and Nadal-Ginard B. (1983). Reversibility of muscle differentiation in the absence of commitment: analysis of a myogenic cell line temperature-sensitive for commitment. Cell **45:** 281–283.

Noelle R.J., Krammer P.H., Ohara J., Uhr J.W., and Vitetta E.S. (1984). Increased expression of Ia antigens on resting B cells: an additional role for B-cell growth factor. Proc. Natl. Acad. Sci. USA **81:** 6149–6153.

Noelle R.J., Kuziel W.A., Maliszewski C.R., McAdams E., Vitetta E.S., and Tucker P.W. (1986). Regulation of the expression of multiple class II genes in murine B cells by B cell stimulatory factor-1 (BSF-1). J. Immunol. **137:** 1718–1723.

O'Garra A., Warren D.J., Holman M., Popham A.M., Sanderson C.J., and Klaus G.G.B. (1986). Interleukin 4 (B-cell growth factor II/eosinophil differentiation factor) is a mitogen and differentiation factor for preactivated murine B lymphocytes. Proc. Natl. Acad. Sci. USA **83:** 5228–5232.

Ohara J., and Paul W.E. (1987). Receptors for B-cell stimulatory factor-1 expressed on cells of haematopoietic lineage. Nature **325:** 537–540.

Paavonen T., Andersson L.C., and Adlercreutz H. (1981). Sex hormone regulation of in vitro immune response. J. Exp. Med. **154:** 1935–1945.

Palacios R., Sideras P., and von Boehmer H. (1987). Recombinant interleukin 4/BSF-1 promotes growth and differentiation of intrathymic T cell precursors from fetal mice in vitro. EMBO J. **6:** 91–95.

Park L.S., Friend D., Grabstein K., and Urdal D.L. (1987). Characterization of the high-affinity cell-surface receptor for murine B-cell-stimulating factor 1. Proc. Natl. Acad. Sci. USA **84:** 1669–1673.

Paul W.E., and Ohara J. (1987). B-cell stimulatory factor-1/interleukin-4. Annu. Rev. Immunol. **5:** 429–459.

Polla B.S., Poljak A., Ohara J., Paul W.E., and Glimcher L.H. (1986). Regulation of class II gene expression: analysis in B cell stimulatory factor 1-inducible murine pre-B cell lines. J. Immunol. **137:** 3332–3337.

Ponzetto C., and Wolgemuth D.J. (1985). Haploid expression of a unique c-*abl* transcript in the mouse male germ line. Mol. Cell. Biol. **5:** 1791–1794.

Rabin E.M., Ohara J., and Paul W.E. (1985). B-cell stimulatory factor 1 activates resting B cells. Proc. Natl. Acad. Sci. USA **82:** 2935–2939.

Rigby P.W.J., Dieckmann M., Rhodes C., and Berg P. (1977). Labeling deoxyribonucleic acid to high specific activity in vitro by nick translation with DNA polymerase I. J. Mol. Biol. **113:** 237–251.

Roehm N.W., Liebson H.J., Zlotnick A., Kappler J., Marrack P., and Cambier J.C. (1984). Interleukin-induced increase in Ia expression by normal mouse B cells. J. Exp. Med. **160:** 679–694.

Rothman P., Lutzker S., Cook W., Coffman R., and Alt F.W. (1988). Mitogen plus interleukin 4 induction of C$\epsilon$ transcripts in B lymphoid cells. J. Exp. Med. **168:** 2385–2389.

Ryder K., Lau L.F., and Nathans D. (1988). A gene activated by growth factors is related to the oncogene v-*jun*. Proc. Natl. Acad. Sci. USA **85:** 1487–1491.

Sanger F. (1977). DNA sequencing with chain-terminating inhibitors. Proc. Natl. Acad. Sci. USA **74:** 5463–5467.

Schackleford G.M., and Varmus H.E. (1987). Expression of the proto-oncogene *int*-1 is restricted to postmeiotic male germ cells and the neural tube of mid-gestational embryos. Cell **50:** 89–95.

Snapper C.M., and Paul W.E. (1987). B cell stimulatory factor-1 (interleukin 4) prepares resting murine B cells to secrete IgG1 upon subsequent stimulation with bacterial lipopolysaccharide. J. Immunol. **139:** 10–17.

Snapper C.M., Hornbeck P.V., Atasoy U., Pereira G.M.B., and Paul W.E. (1988). Interleukin 4 induces membrane Thy-1 expression on normal murine B cells. Proc. Natl. Acad. Sci. USA **85:** 6107–6111.

Sorrentino V., McKinney M.D., Giorgi M., Geremia R., and Fleissner E. (1988). Expression of cellular protooncogenes in the mouse male germ line: a distinctive 2.4-kilobase *pim*-1 transcript is expressed in haploid postmeiotic cells. Proc. Natl. Acad. Sci. USA **85:** 2191–2195.

Strich G., Petze J.E., Silva de Sa, M.F., and Rebar R.W. (1985). The effects of thymus-derived peptides on hypothalamic LRF and pituitary gonadotropin content in prepubertal congenitally athymic nude mice and their normal heterozygous littermates. J. Reproduc. Immunol. **7:** 351–359.

Strickland S., Smith K.K., and Marotti K.R. (1980). Hormonal induction of differentiation in teratocarcinoma stem cells: generation of parietal endoderm by retinoic acid and dibutyryl cAMP. Cell **21:** 347–355.

Wolfes H., Kogawa K., Millette C.F., and Cooper G.M. (1989). Specific expression of nuclear proto-oncogenes before entry into meiotic prophase of spermatogenesis. Science **245:** 740–743.

Wolgemuth D.J., Ginzang-Ginsberg E., Engelmyer E., Gavin B.J., and Ponzetto C. (1985). Separation of mouse testis cells on a Celsep apparatus and their usefulness as a source of high molecular weight DNA and RNA. Gam. Res. **12:** 1–10.

Wolgemuth D.J., Engelmyer E., Duggal R.N., Ginzang-Ginsberg E., Mutter G.L., Ponzetto C., Viviano C., Zakeri Z.F. (1986). Isolation of a mouse cDNA coding for a developmentally regulated testis-specific transcript containing homeo box homology. EMBO J. **5:** 1229–1235.

Wolgemuth D.J., Viviano C.M., Ginzang-Ginsberg E., Frohman M.A., Joyner A.L., and Martin G.R. (1987). Differential expression of the mouse homeobox-containing gene *Hox*-1.4 during male germ cell differentiation and embryonic development. Proc. Natl. Acad. Sci. USA **84:** 5813–5817.

